# Persistent Neutrophil Infiltration and Unique Ocular Surface Microbiome Typify Dupilumab–Associated Conjunctivitis in Patients with Atopic Dermatitis

**DOI:** 10.1016/j.xops.2023.100340

**Published:** 2023-05-29

**Authors:** VijayKumar Patra, Nora Woltsche, Urban Cerpes, Danijela Bokanovic, Maria Repelnig, Aaroh Joshi, Isabella Perchthaler, Manuela Fischl, Marc Vocanson, Natalie Bordag, Marija Durdevic, Johannes Woltsche, Franz Quehenberger, Franz Legat, Andreas Wedrich, Jutta Horwath-Winter, Peter Wolf

**Affiliations:** 1 Department of Dermatology, Medical University of Graz, Graz, Austria; 2 Centre International de Recherche en Infectiologie, Institut National de la Santé et de la Recherche Médicale, U1111, Université Claude Bernard Lyon 1, Centre National de la Recherche Scientifique, UMR5308, École Normale Supérieure de Lyon, Université de Lyon, Lyon, France; 3 Department of Ophthalmology, Medical University of Graz, Graz, Austria; 4 Computational Bioanalytics, Center for Medical Research, Medical University of Graz, Graz, Austria; 5 Institute of Pathology, Medical University of Graz, Graz, Austria; 6 Theodor Escherich Laboratory for Medical Microbiome Research, Medical University of Graz, Graz, Austria; 7 BioTechMed Graz, Graz, Austria

**Keywords:** Dupilumab, Atopic dermatitis, Microbiome, Cytokines, Neutrophils

## Abstract

**Objective:**

To elucidate the pathogenesis of dupilumab (Dupixent®)–associated ocular surface disease (DAOSD).

**Design:**

Prospective single-center cohort study.

**Subjects:**

Twenty patients with moderate-to-severe atopic dermatitis (AD) who received dupilumab and 10 age- and sex-matched healthy controls were enrolled in the study.

**Methods:**

The study cohort underwent a thorough slit-lamp and entire-body dermatologic examination. Conjunctival swabs and smears were collected at baseline, 4 and 16 weeks after treatment initiation, and during the conjunctivitis episode. To analyse the ocular surface microbiome, 16S ribosomal RNA sequencing was performed, smears were hematoxylin and eosin stained, and serum cytokines were measured by using a multiplex immunobead assay.

**Main Outcome Measures:**

Composition of ocular surface microbiome and cellular component as well as serum cytokine levels.

**Results:**

Six of the 20 patients with AD developed DAOSD after dupilumab initiation; these patients responded after a delay to treatment as quantified by Eczema Area and Severity Index and Investigator’s Global Assessment score. Conjunctival smears showed massive neutrophilic infiltration and serum analysis revealed increased systemic levels of neutrophil-priming proinflammatory cytokines, in particular interleukin-1β and tumor necrosis factor α, in patients with DAOSD compared with those without it. The ocular surface microbiome of patients with DAOSD was characterized by a diverse and persistent microbial colonization, particularly by *Acetobacter aceti*. In contrast, microbial diversity decreased in patients with AD without DAOSD after the initiation of dupilumab treatment, especially the abundance of *Staphylococcus aureus*. In vitro experiments substantiated the potential role of the microbiome, showing increased growth of *A. aceti* and decreased growth of *S. aureus* in presence of dupilumab.

**Conclusions:**

Persistent neutrophilic infiltration and a unique microbial landscape on the ocular surface associated with elevated levels of systemic proinflammatory cytokines typify DAOSD.

**Financial Disclosure(s):**

Proprietary or commercial disclosure may be found after the references.

Atopic dermatitis (AD) is a chronic, recurrent, inflammatory disease characterized by pruritus, xerosis, and eczematous skin lesions. This disease has a prevalence of approximately 2% to 10% in the adult population.^1^ Up to 50% of the patients present with moderate-to-severe forms of the disease, which significantly limits their quality of life.^1^ Dupilumab (Dupixent®) is a fully human IgG4 monoclonal antibody targeting the interleukin (IL) 4 receptor-α subunit of IL-4 and IL-13. This antibody has been approved in many countries to treat moderate-to-severe AD requiring systemic treatment in adults^2^. Recently, it was also approved for moderate-to-severe AD requiring systemic treatment in adolescents and for severe AD requiring systemic treatment in children aged ≥ 6 months.^2^ Randomized, double-masked, placebo-controlled, multinational phase III studies (SOLO I and II, CHRONOS, and CAFÉ) have revealed significant improvement in signs and symptoms in patients who received a loading dose of 600 mg of dupilumab and 16 weeks of treatment with 300 mg of dupilumab q2w (i.e., once every 2 weeks) either alone or in addition to topical corticosteroids.^3^, ^4^, ^5^ The efficacy of dupilumab has been substantiated in the clinical setting and is now also recommended as a long-term treatment.^6^

However, patients in clinical studies (SOLO I and II, CHRONOS, and CAFÉ) have experienced adverse events when treated with dupilumab, the most common of which is the development of ocular surface disease (5%–28% of patients), particularly conjunctivitis, as compared with 1% to 11% of patients treated with a placebo.^3^, ^4^, ^5^ In clinical settings, as many as 60% of patients have been reported to experience ocular surface adverse events, including blepharitis and keratitis in addition to conjunctivitis.^6^ Interestingly, these high percentages of ocular surface adverse events have not been reported in patients with bronchial asthma, chronic rhinosinusitis with nasal polyposis, eosinophilic esophagitis, or prurigo nodularis for whom dupilumab has also been approved. Thus, these ocular surface adverse events may be considered AD-specific. The dupilumab–associated ocular surface disease (DAOSD) may be so severe that this otherwise well-tolerated and highly effective treatment needs to be reduced or discontinued. The pathogenesis of DAOSD has not been fully elucidated, but several hypotheses have been proposed, e.g., an underlying subclinical atopic eye disease,^7^ infestation with Demodex mites,^8^ ocular immunodeficiency leading to local infections and upregulation of proinflammatory molecules,^6^^,^^9^ a deficit in tear production,^10^ decreased mucus production,^11^ a deficiency in conjunctival goblet cells,^12^ drug-induced eosinophilia,^13^^,^^14^ decreased bioavailability of dupilumab on the ocular surface,^15^ or a Th1 shift associated with atopic keratoconjunctivitis due to a blockade of the Th2 pathway by dupilumab.^16^

In the present study, we investigated the ocular surface microbiome, cellular components, and systemic cytokine levels in dupilumab-treated patients with AD with and without DAOSD. We found massive and persistent neutrophilic infiltration in conjunctival smears of patients with DAOSD. Intriguingly, neutrophilic inflammation was linked to elevated levels of IL-1β and tumor necrosis factor α (TNF-α) in serum, and a persistent and differential ocular microbial landscape was observed in patients with DAOSD.

## Methods

### Patient Characteristics

This study enrolled patients aged > 18 years with moderate-to-severe AD who were scheduled for systemic treatment with dupilumab. Patients were excluded if they were allergic to 0.4% oxybuprocaine hydrochloride eye drops used for topical ocular anesthesia, patients with ocular diseases, such as glaucoma, who required regular use of eye drops (except artificial tear eye drops), patients with an active ocular surface microbial inflammation or active allergic conjunctivitis at baseline, patients wearing contact lenses, and patients who had undergone ophthalmic surgery in the past 6 months. The study described here conformed to the principles of the Declaration of Helsinki. Informed consent was obtained from all study participants and controls. Approval was obtained from the Ethics Committee of the Medical University of Graz (ethical application number 31-379 ex 18/19).

### Patient Treatment

We asked the patients for clinical history of preexisting ocular disease, obtained conjunctival smears and swabs, and performed a thorough slit-lamp examination and a full-body dermatologic examination (including completing the AD scores Eczema Area and Severity Index [EASI] and Investigator's Global Assessment [IGA]) at baseline, before initiating dupilumab treatment (baseline), 4 weeks after treatment initiation (visit 1m), upon DAOSD development (visit conj), and upon ceasing treatment after 16 weeks (visit 4m). Treatment included a loading dose of 600 mg followed by a dupilumab treatment of 300 mg q2w for 16 weeks. The control group included 10 healthy subjects without AD; conjunctival smears and swabs were obtained from these subjects, and a thorough slit-lamp examination was performed at a single time point.

### Clinical Dermatological Scoring

The EASI and IGA scoring was completed at each visit for each patient by 1 out of a group of 3 experienced dermatologists (U.C., D.B., and M.R.). These established scores quantify the severity of the disease, where the EASI allows a ranking from 0 to 72, and IGA, from 0 to 4.^17^

### Patient Conjunctival Sampling

#### Conjunctival Smears and Conjunctival Bacterial Swabs

Under local anesthesia of the ocular surface with 0.4% oxybuprocaine hydrochloride eye drops, the inferior fornix of the right eye was gently wiped with a plastic loop. The material was then placed on a glass slide and air-dried. Bacterial swabs were taken from the inferior fornix of the right eye by carefully wiping with ESwab (Copan Diagnostics).

### Histologic Analysis

Conjunctival smears on the glass slides were stained with hematoxylin and eosin. Cellular infiltrate was visually evaluated and quantified by 2 independent investigators (N.W. and V.P.) at 5 randomly selected sites per sample under the microscope at 40× magnification.

### Microbial DNA Extraction

Total DNA was isolated from conjunctival bacterial swabs by both mechanical and enzymatic lysis. Samples were thawed and transferred to sterile MagNA Lyser Green Bead Tubes (Roche) and bead-beaten twice to achieve mechanical lysis at 6000 rpm for 30 seconds in a MagNA Lyser instrument (Roche). Unused swabs and unused buffer tubes without swabs served as negative controls for sample collection and DNA isolation. Samples were subjected to enzymatic lysis by incubating them with 2.5-μl lysozyme (100 mg/ml: Carl Roth) at 37° C for 1 hour. Further processing was performed using a QIAamp DNA microbiome kit (#51704, Qiagen) according to the manufacturer's instructions. Total DNA was eluted in 35 μl of the AE buffer included in the kit. Total DNA was stored at −20° C until polymerase chain reaction (PCR) amplification. All procedures were performed under sterile conditions in a laminar flow unit.

### 16s Microbiome Sequencing

16s ribosomal RNA library preparation, quantification, and sequencing were performed as recently published by Klymiuk et al.^18^ Briefly, 5 μl of total DNA were used for PCR amplification with the target-specific primers 27F 5' - AGAGTTGATCCTGGCTCAG - 3' and R357 5' - CTGCTGCCTYCCGTA - 3' (MWG-Biotech AG, Germany ). The PCR reaction consisted of an initial denaturation at 95° C for 3 minutes, followed by 32 cycles of denaturation at 95° C for 45 seconds, annealing at 55° C for 45 seconds, and extension at 72° C for 1 minute. The final extension was at 72° C for 7 minutes, and PCR reactions were performed in triplicate. The triplicate reactions were pooled, and 5 μl were visualized on a 1% agarose gel to verify amplification success. The normalization, indexing, pooling of individual PCR products, and purification of the final library were performed as described in Klymiuk et al.^18^ The library was quantified using the QuantiFluor ONE dsDNA kit (Promega) according to the manufacturer's instructions and visualized on an Agilent 2100 Bioanalyzer (Agilent Technologies) using a high-sensitivity DNA assay according to the manufacturer's instructions. The 6 pM library was sequenced on a MiSeqII desktop sequencer (Illumina) using version 3 chemistry for 600 cycles. For data analysis, 20% PhiX control DNA and FastQ files were used.

### 16s Bioinformatic Analysis

Illumina paired-end raw sequencing data were first checked for quality and any present sequencing adapters. Quantitative Insights into Microbial Ecology (QIIME2 version 2022.2),^19^ a bioinformatic pipeline integrated in the local galaxy instance (https://galaxyproject.org/) hosted on the MedBioNode HPC cluster of the Medical University Graz in Austria (https://galaxy.medunigraz.at/), was used to analyse the final sequence files. A total of 1 701 562 raw sequence reads were quality-filtered, denoised, dereplicated, merged, and checked for chimeras using DADA2 denoise pipeline^20^ with optimized parameters p-trunc-len-f: 270, p-trunc-len-r: 230, p-trim-left-f: 20, p-trim-left-f: 15, and p-max-ee: 2.0 as implemented in QIIME2 microbiome bioinformatics platform. The taxonomic assignment of the DADA2 representative sequence set (amplicon sequence variant [ASV]) was accomplished with the QIIME2 sklearn-based classifier, comparing against the SILVA ribosomal RNA database Release 138 at 99% identity.^21^ All further downstream statistical data analyses, including alpha and beta diversity, were conducted with the R version 4.1.0 program for statistical computing (https://www.R-project.org)^22^ accompanied with the packages vegan, ggplot2, and psych or Microbiome analyst.^23^^,^^24^ Data were uploaded to European Nucleotide Archive, supported by EMBL-EBI, and assigned the primary accession number PRJEB57775.

### Serum Cytokine Analysis

A ProcartaPlex human cytokine 65-plex kit purchased from ThermoFisher Scientific was used to measure the serum concentration of 65 different cytokines. Following the manufacturer’s instructions, 25 μl of undiluted frozen serum samples were processed in 96-well plates. Standard curves for each analyte were generated using the reference analyte concentration supplied by the manufacturer. The measurement was performed on a calibrated Bio-Plex 200 system (Bio-Rad) in combination with Bio-Plex Manager software (version 6.1, Bio-Rad). Each sample was measured in duplicate, and the cytokine concentration was calculated from the standard curve using 5PL curve fitting. Raw measurements were subjected to nonparametric longitudinal analysis. False discovery rates < 0.05 were statistically significant. The R package nparLD (version 2.1) was used to perform calculations. Effect sizes were calculated on the logarithmic scale (log2 fold change). Groups effects transformed to the fluorescence intensity (FI) scale by exponentiation, resulting in percentages. The standard errors of these effects were calculated by applying the delta method.

### Bacterial Culture

Commercially available freeze-dried bacterial strains were reconstituted according to supplier’s instructions. The reconstituted bacteria were grown for 18 hours in the appropriate media. Optical density at 600 nm was adjusted to ∼ 1 for *Staphylococcus aureus* (ATCC/ BAA-17717) in tryptic soy broth and 0.5 for *Acetobacter aceti* (DSMZ- 3508) in yeast peptone mannitol medium. Aliquots were stored at −80° C until further use.

### Microplate Growth Curve Assay

Thawed aliquots of bacterial suspension were serially diluted (1:10) 2 times (10^−2^). Subsequently, 1 ml of the 10^−2^ dilution was further diluted in 9 ml of an appropriate medium containing 2.77 mg/l, 5.55 mg/l, and 11.11 mg/l dupilumab (Dupixent, Sanofi Genzyme) to obtain a final concentration of 2.5 mg/l, 5 mg/l, and 10 mg/l dupilumab, respectively. Two hundred uL of each concentration and the control bacterial suspension (without dupilumab) were added to the sterile Nunc 96 well microtiter plates (Thermo Fisher Scientific), and the optical density at 600 nm was measured on regular intervals for 48 hours using SPECTROstar omega (BMC labtech).

### Statistical Analysis

Statistical analyses was performed in GraphPad Prism (version 9.3, GraphPad Software) or by using R 4.1.0 program for statistical computing (https://www.R-project.org).^22^ Statistical tests used are specified in the figure legends. For the microbiome analysis, analysis of similarities was performed by using R to determine the significance between groups in nonmetric multidimensional scaling.

## Results

### Patients and DAOSD

A total of 20 patients (13 men and 7 women; mean age, 49; range, 20–87 years) with moderate-to-severe AD treated with dupilumab and 10 healthy controls without AD (5 men and 5 women; mean age, 34; range, 25–57 years) were enrolled in the study. Overall, 6 (30%) of the 20 patients with AD developed DAOSD within a median time of 54 days (range, 28–103 days) after the initiation of dupilumab treatment. Occurrence of DAOSD in our cohort is in line with current reports.^25^ Moreover, a recent report with a large cohort showed that AD severity and conjunctivitis at baseline are not risk factors.^25^

All 6 patients displayed blepharoconjunctivitis, and 2 also developed keratitis. The patients with blepharoconjunctivitis had swollen, erythematous, and sometimes scaly eyelids, hyperemia of the conjunctiva, and mucous secretion with symptoms, including foreign body sensation, burning, itching, and epiphora.

These 6 patients were treated with topical lubricants and eyelid hygiene, or either with calcineurin inhibitor (0.03% tacrolimus ointment applied twice daily to eyelids and lower fornices), or combination of mast cell stabilizer/antihistamine; all responded satisfactorily. One of the 2 patients with keratitis had a small superficial corneal infiltrate that resolved after treatment with topical lubricants, antibiotics, and corticosteroids, whereas the other patient had superior limbitis with lesions resembling Horner-Trantas spots that responded to topical lubricants, corticosteroids, and a mast cell stabilizer/antihistamine combination. Dupilumab–associated ocular surface disease resolved in all of the 6 patients during the study period (visit 4m), and none of these had to discontinue dupilumab. Detailed characteristics of the patients and controls are shown in Table 1.

**Table 1:** Characteristics of Patients and Controls

	Patients with AD without DAOSD	Patients with AD and DAOSD	*P* Value‡	Controls
Count (%)	14 (70)	6 (30)		10
Age (yrs, median)∗	45	37	0.74	30
Gender:				
Male (count, %)	9 (64)	4 (67)		5 (50)
Female (count, %)	5 (36)	2 (33)		5 (50)
Male/female (count)†			> 0.99	
History of ocular surface disease (count, %)†	6 (43)	3 (50)	> 0.99	0
Topical lubricants at baseline(count, %)	4 (29%)	2 (33%)	> 0.99	0
Asthma bronchial (count, %)†	4 (29)	2 (33)	> 0.99	0
Visit 1 - baseline				
No. of patients (count, %)	14 (100)	6 (100)		10 (100)
EASI (median)	33.2	27.2	0.43	NA
IGA (median)	4	4	0.58	NA
Visit 2 – 1 mo				
No. of patients (count, %)	11 (79)	5 (83)		NA
EASI (median)	13.9	14.6	0.46	NA
IGA (median)	3	3	0.91	NA
Visit 3 – 4 mos				
No. of patients (count, %)	12 (86)	6 (100)		NA
EASI (median)	8.5	10.9	0.35	NA
IGA (median)	2	3	0.91	NA
Visit DAOSD				
No. of patients (count, %)	NA	6 (100)		NA
Interval between visit 1 and visit DAOSD (days [median], range)	NA	53.5 (28–103)		NA
EASI (median)	NA	11		NA
IGA (median)	NA	3		NA
Conjunctivitis (count, %)	NA	6 (100)		NA
Keratitis (count, %)	NA	2 (33)		NA
Blepharitis (count, %)	NA	6 (100)		NA

AD = atopic dermatitis; DAOSD = dupilumab–associated ocular surface disease;

EASI = Eczema Area and Severity Index; IGA = Investigator Global Assessment; NA = not applicable.

∗ Wilcoxon rank sum test.

† Fisher exact test for count data.

‡ *P* values are shown for comparisons between patients with AD and DAOSD vs. without DAOSD.

### Treatment Response of AD

**Figure 1:**
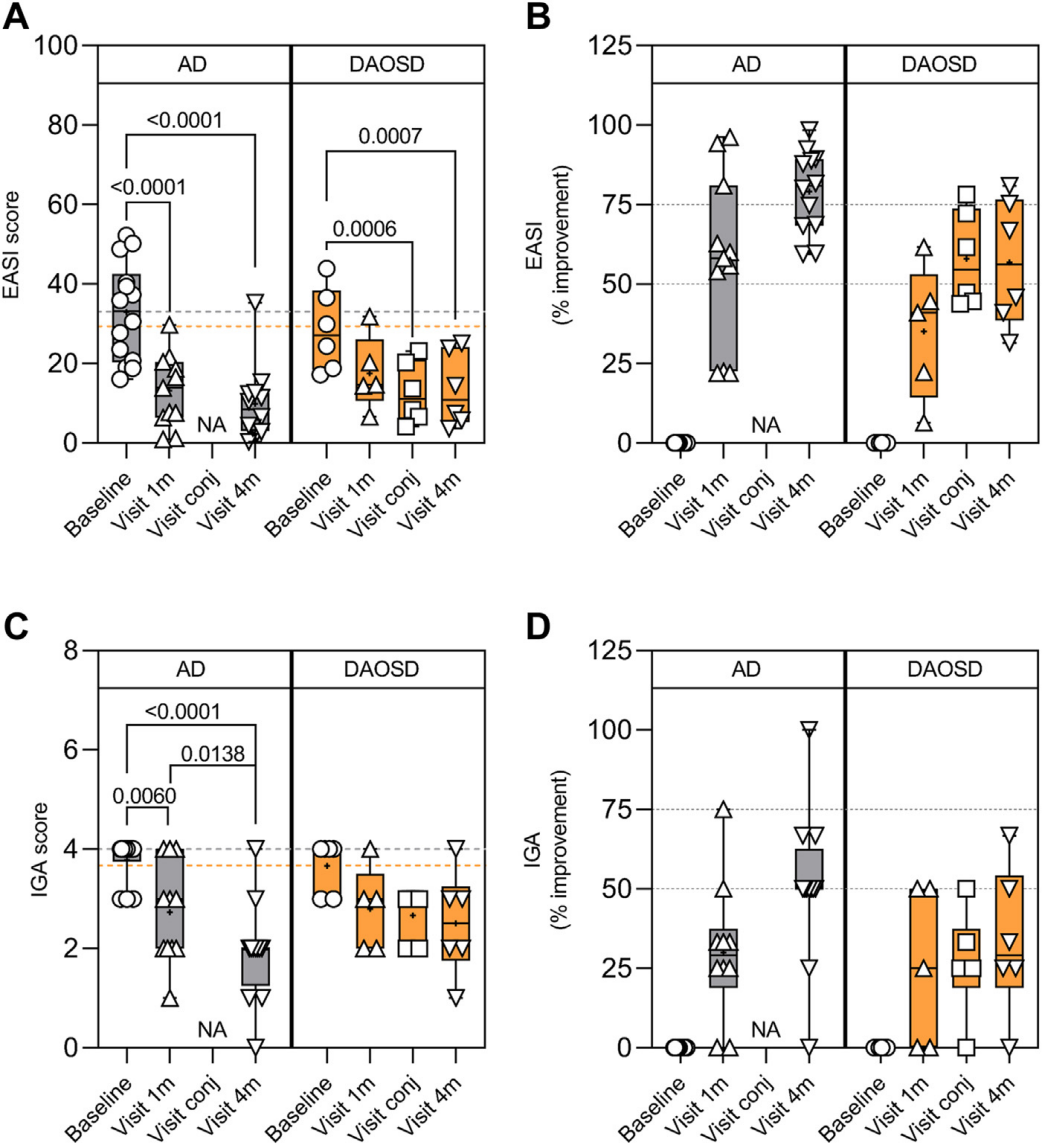
Clinical outcome in patients with atopic dermatitis (AD) treated with dupilumab. **A,** Eczema Area and Severity Index (EASI) score, (**C**) Investigator Global Assessment (IGA) score, (**B**) percentage of improvement in EASI, and (**D**) IGA during different visits of patients with AD and dupilumab–associated ocular surface disease (DAOSD). Data are presented in box and whisker plots showing minimum, maximum, and median values; a diminutive (+) indicates mean values, () gray dotted lines indicate the mean value at baseline in AD, and () orange dotted lines indicate the mean value at baseline in DAOSD. N = 6–14. One-way analysis of variance was used for statistical testing. NA = not applicable.

The median EASI score of 20 patients at baseline was 30.2 (range, 16–52.2). No significant difference in baseline characteristics was seen when comparing the 6 DAOSD patients with the remaining 14 patients with AD (Table 1). The median EASI score of the 14 patients who did not develop DAOSD was 33.2 (range, 16–52.2) at baseline and decreased to 13.9 (range, 0.9–29.7) after 1 month and to 8.5 (range, 0.3–35.30) 4 months after the initiation of dupilumab. This decrease was statistically significant after both 1 month (*P* < 0.001) and 4 months (*P* < 0.001) as compared with baseline (Fig 1A). Three (27.2%) of 11 patients achieved an EASI75 after 1 month, which increased to 8 (66.6%) of 12 patients after 4 months (Fig 1B). In patients with DAOSD, the median EASI decreased from 27.1 (range, 17.2–43.9) at baseline, to 14.6 (range, 6.6–31.8) after 1 month (not significant) and to 10.9 (range, 3.6–25.0) after 4 months (*P* < 0.001); at the time of conjunctivitis (*P* < 0.001), the median EASI was 11 (range, 4.1–23.1). No patients reached EASI75 after 1 month, whereas 1 (16.7%) of the 6 patients reached EASI75 during the conjunctivitis episode, and 2 (33.3%) patients reached this after 4 months (Fig 1B). Likewise, the EASI and IGA response was also better in patients who did not develop DAOSD. Investigator’s Global Assessment could only be significantly reduced in patients with AD without DAOSD, but not in patients with DAOSD (Figure 1C, D).

Patients initiating dupilumab in summer or early fall developed DAOSD more frequently, but overall, the seasonal distribution did not reach statistical significance (*P* = 0.12) (Fig S1, available at www.ophthalmologyscience.org). Taken together, our data show a delayed clinical response to dupilumab in patients with DAOSD as compared with patients without DAOSD.

### Massive Neutrophilic Infiltration in DAOSD

**Figure 3:**
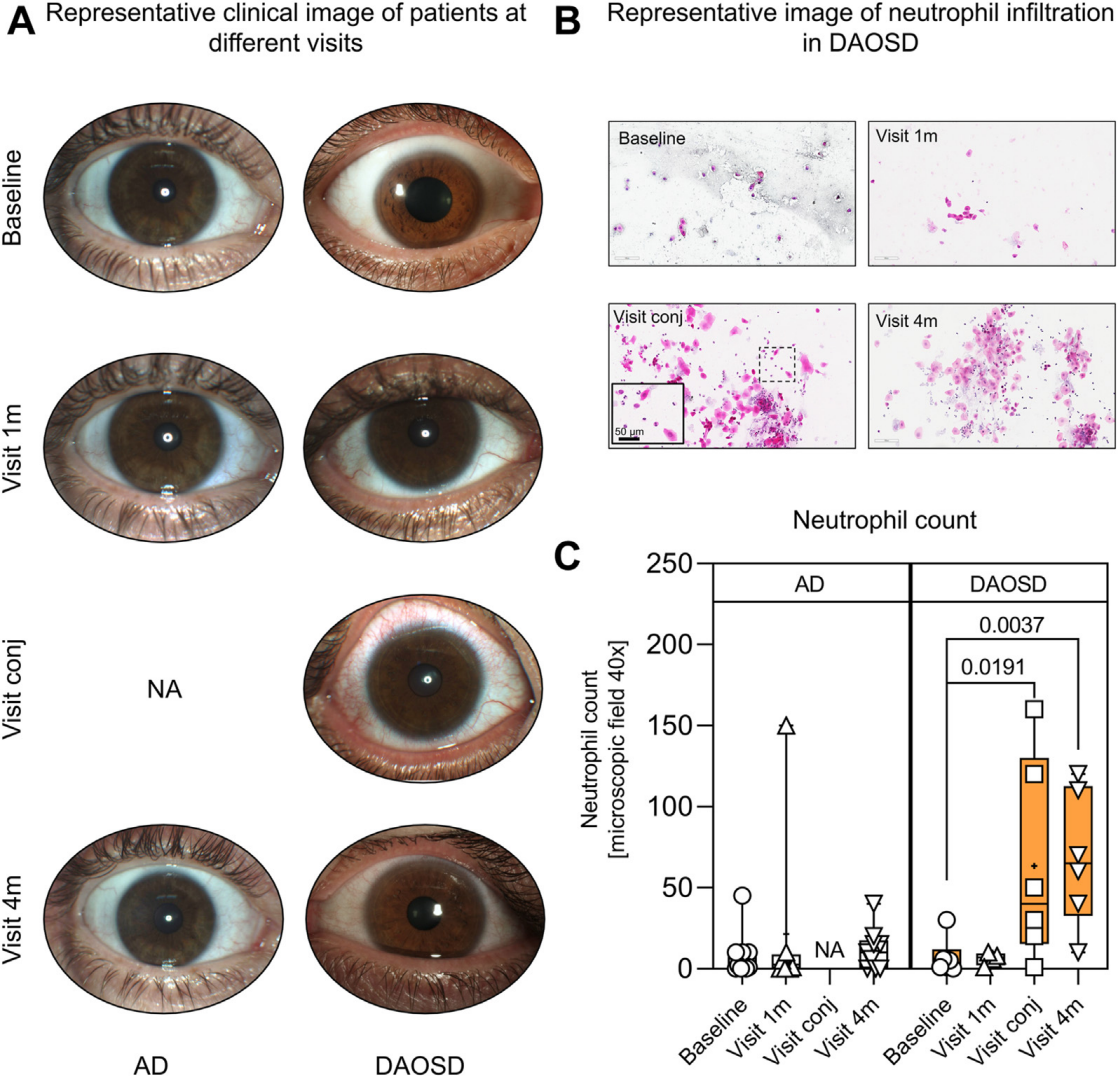
Cellular infiltrate in ocular surface after dupilumab treatment. **A,** Representative clinical images from patients with atopic dermatitis (AD) and dupilumab–associated ocular surface disease (DAOSD) taken during different visits. **B,** Histologic image of cellular infiltrate at different time points in DAOSD development. **C,** Quantitative data of cellular infiltrate are presented in box and whisker plots showing minimum, maximum, and median values; a diminutive (+) indicates mean values. N = 6–14. One-way analysis of variance was used for statistical testing. NA = not applicable.

Representative clinical photographs of DAOSD are depicted in Figure 3A. Hematoxylin and eosin stainings of conjunctival smears taken from the inferior fornix revealed a massive cellular infiltrate composed mainly of neutrophils, as shown in a representative image of a patient with DAOSD (Fig 3B). The neutrophilic infiltration reached statistical significance during the conjunctival episode (*P* = 0.0191) and 4 months (*P* = 0.0037) after the initiation of dupilumab as compared with baseline (Fig 3C), whereas we did not detect significant numbers of neutrophils after 1 month.

### Unique Ocular Microbial Landscape in Patients with DAOSD

Atopic dermatitis and conjunctivitis are associated with changes in microbial composition and an increased abundance of pathogenic microbes.^26^^,^^27^ To investigate the association between the microbiome and DAOSD, we collected swabs from the inferior fornix of the eye and subjected these to microbiome sequencing as described in the Methods section. Bacterial 16S amplicon sequencing (V1–V2) of the samples yielded 2 332 922 reads after combining all sequence data sets. After preprocessing, we obtained 1 701 562 reads (73%), giving an average of 47 610.65 ± 17 137.13 reads per sample. All samples reached a plateau at 7000 reads/samples for the rarefaction, indicating sufficient sequencing depth. Reads were assigned to 3370 ASVs with an average length of 307.6 ± 17.01 bases. Due to the low number of reads, 2 samples were excluded from further analysis.

**Figure 4:**
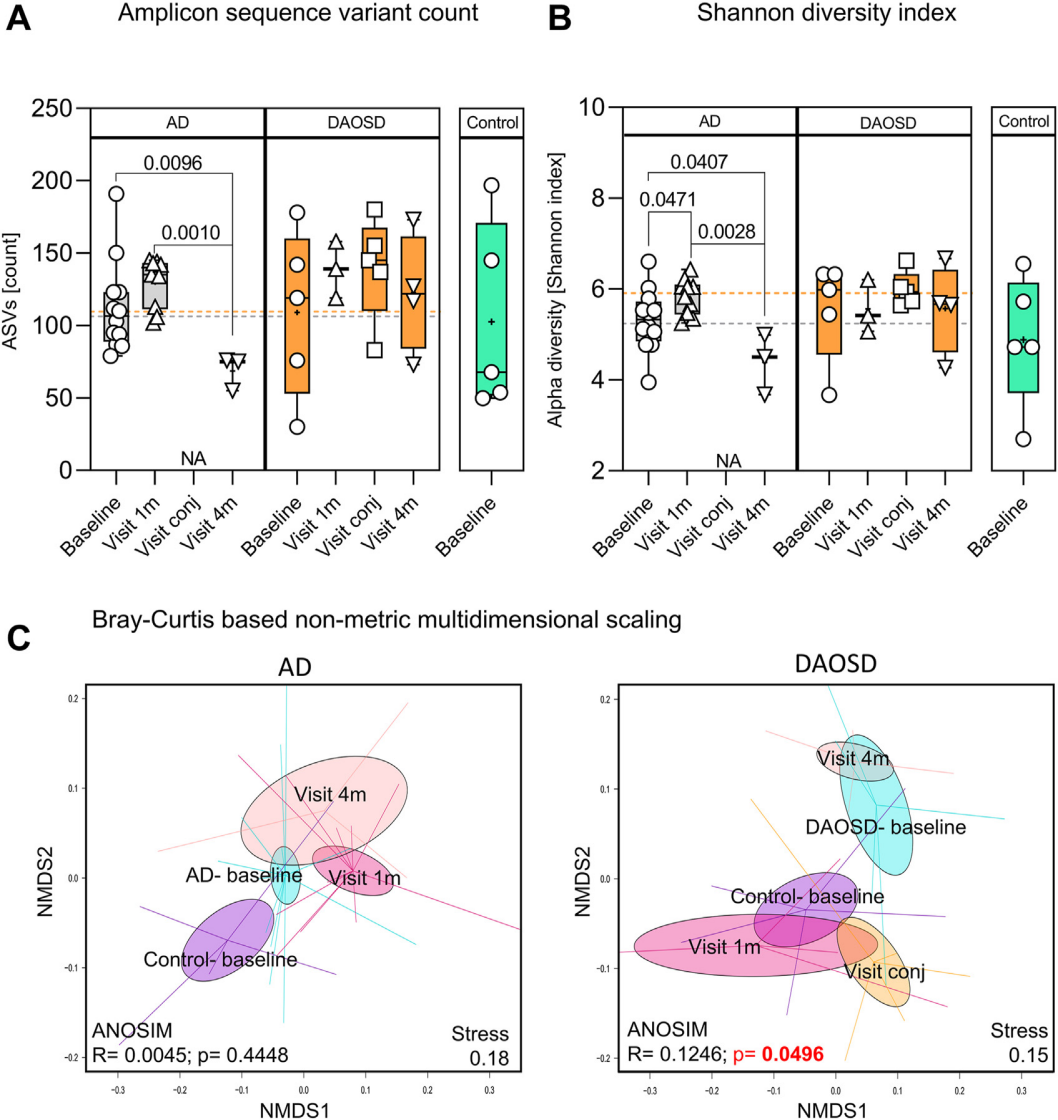
Ocular surface microbiome after dupilumab treatment. Box plots indicate the amplicon sequence variants counts (**A**) and alpha diversity (Shannon index, **B**) obtained from sequence analysis from eye swabs. Data are presented in box and whisker plots showing minimum, maximum, and median values; a diminutive (+) indicates mean values, () gray dotted lines indicate the mean value at baseline in atopic dermatitis (AD), and () orange dotted lines indicate the mean value at baseline in dupilumab–associated ocular surface disease (DAOSD). N = 3–14. One-way analysis of variance was used for statistical testing. **C,** Bray-Curtis dissimilarity-based nonmetric multidimensional scaling plots for AD and DAOSD are shown. Ellipses shown are based on standard error of the mean. Statistical analysis was performed by using analysis of similarities method. ANOSIM = analysis of similarities; ASVs = amplicon sequence variants; NMDS = non-metric multidimensional scaling; NA = not applicable.

In the AD group, we observed a significant decrease in the number of ASVs after 4 months as compared with baseline (*P* = 0.0096) and after 1 month (*P* = 0.001). We found no statistically significant correlation between month 1 and baseline data (*P* = 0.1148). However, in patients with DAOSD, we found no significant difference between any time points. Control subjects showed similar median values as patients with AD or DAOSD at baseline (Fig 4A). The bacterial diversity as measured by the Shannon index showed similar results in patients with AD; we observed a significant decrease in diversity 1 month (*P* = 0.0471) and 4 months (*P* = 0.0407) after dupilumab treatment as compared with baseline. In patients with DAOSD, we found no difference between the time points (Fig 4B). Control subjects showed slightly lower median levels of bacterial diversity than patients with AD or DAOSD at baseline (Fig 4B). Interestingly, patients with AD and DAOSD showed similar numbers of ASVs at baseline (Fig 4A), but the diversity was slightly higher in DAOSD patients (Fig 4B). The decrease in the Shannon index correlated with the increased neutrophilic infiltration during the conjunctivitis episode (Fig S2, available at www.ophthalmologyscience.org).

Next, we performed a nonmetric multidimensional scaling analysis based on the Bray-Curtis dissimilarity and analysis of similarities statistical testing between different time points, which yielded an *r* value of 0.0045 and a *P* value of 0.4448 (Fig 4C) in patients with AD. However, in patients with DAOSD, we found significant differences within the clusters at different time points and much larger dissimilarities among bacterial communities, which were significant in the analysis of similarities analysis with an *r* value of 0.1246 and a *P* value of 0.0496.

Overall, our results indicate that patients with AD displayed a significant reduction in ocular bacterial diversity after 4 months. In contrast, the ocular microbial diversity remained persistently high in patients with DAOSD.

### Species-Level Characterization

**Figure 6:**
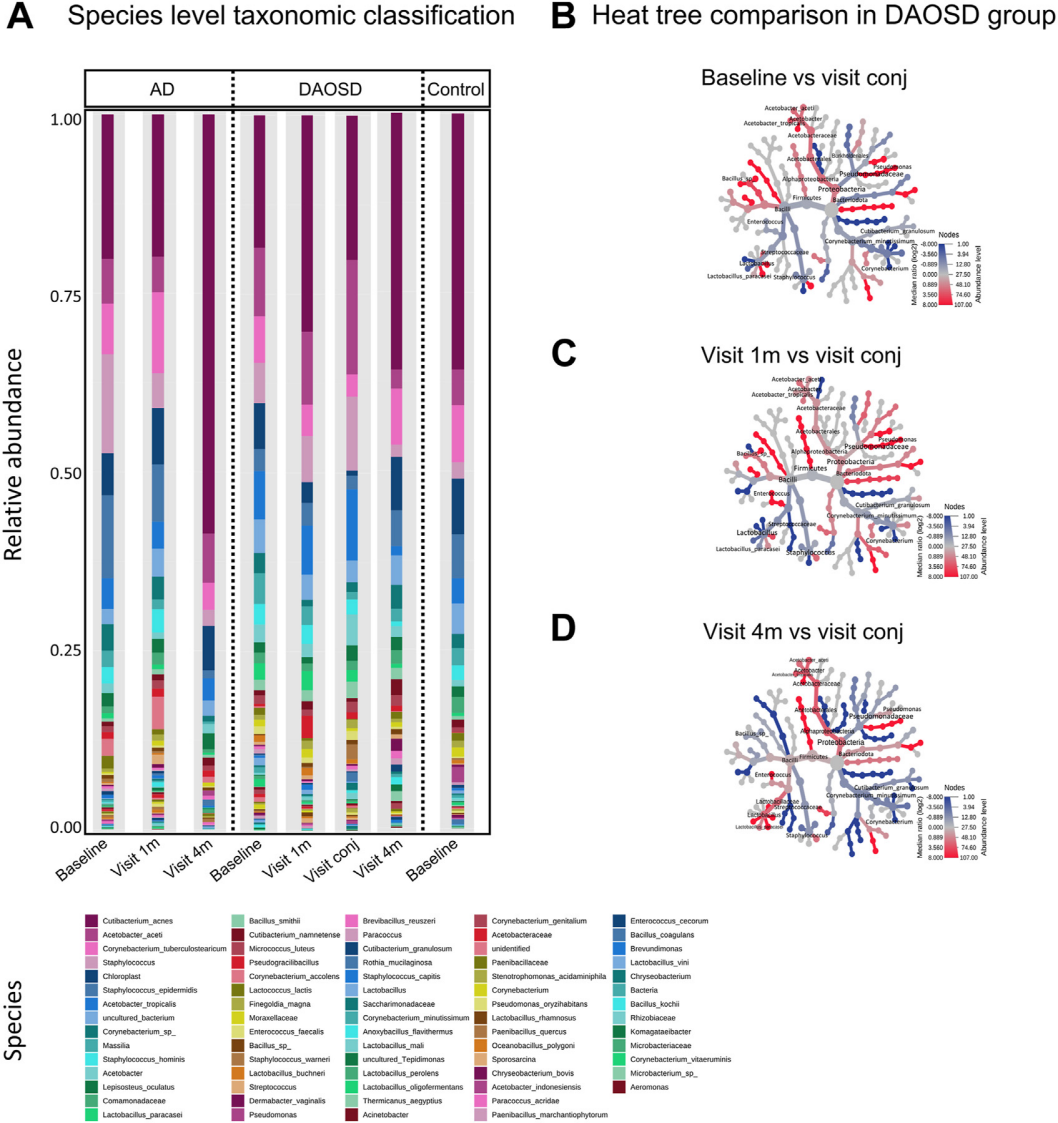
Species-level characterization of ocular surface microbiome. **A,** Stacked bar plot showing relative abundance of bacterial species during different visits in atopic dermatitis (AD), dupilumab–associated ocular surface disease (DAOSD), and control subjects. Heat trees illustrating relative abundances in bacterial species in patients with DAOSD between visit conj (DAOSD development) and baseline (**B**), visit 1m (**C**), and visit 4m (**D**). Color changes denote the difference in log_2_ ratio of median proportions of reads in respective comparisons.

Species-level characterization was performed in our data set as previously described.^28^ In AD without DAOSD, we observed changes in the abundance of certain microbial species belonging to the genera *Staphylococcus, Cutibacterium,* and *Corynebacterium* (Fig 6A). In patients with DAOSD, we observed increased colonization of microbial species belonging to the genera *Acetobacter, Staphylococcus*, and *Cutibacterium* correlating with severity of the disease (Fig 6B).

**Figure 8:**
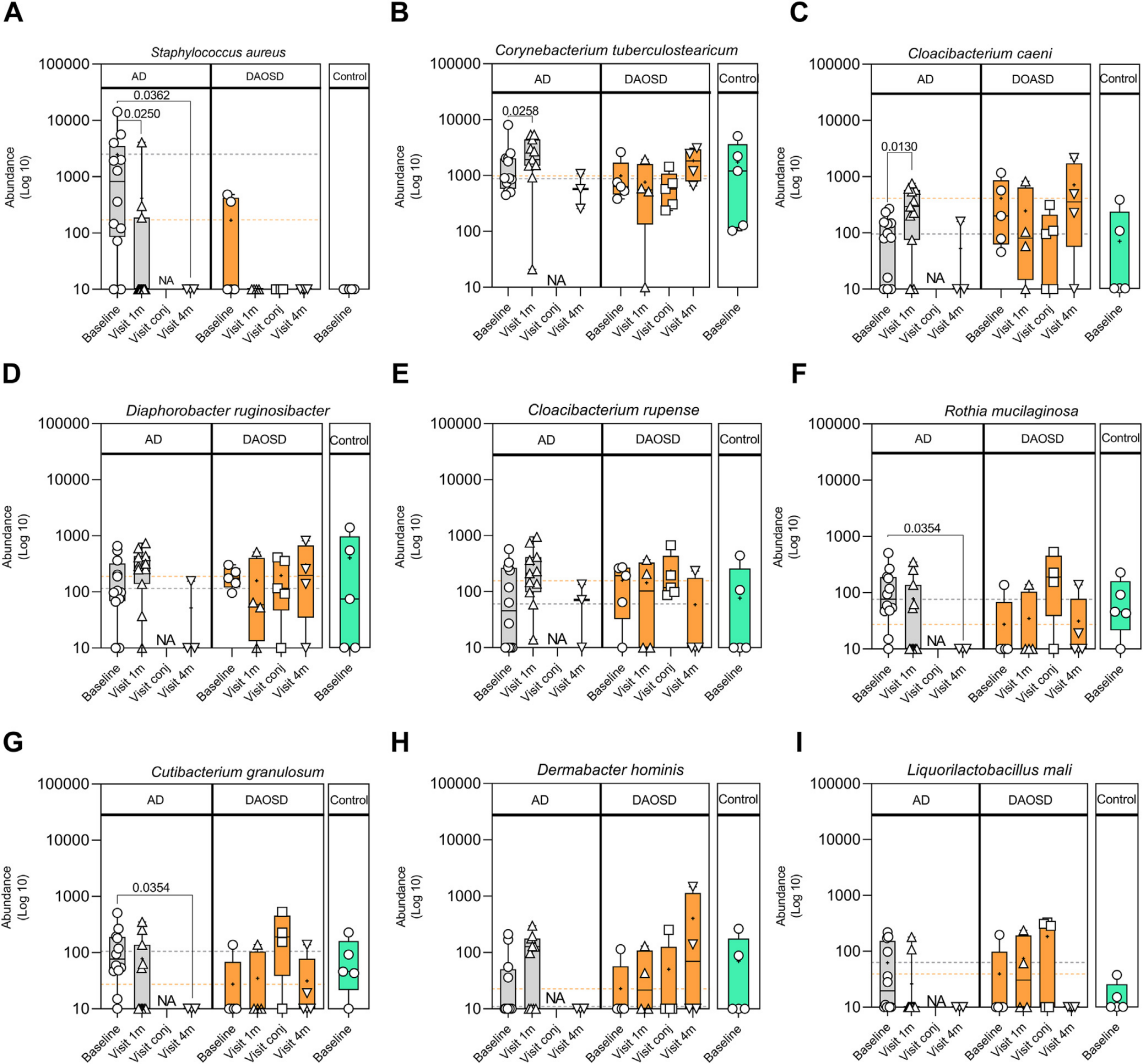
Unique bacterial species in ocular surface of patients with atopic dermatitis (AD). **A–I,** Box plots showing various bacterial species in patients with AD, dupilumab–associated ocular surface disease (DAOSD), and controls. Data are presented in box and whisker plots showing minimum, maximum, and median values; a diminutive (+) indicates mean values, () gray dotted lines indicate the mean value at baseline in AD, and () orange dotted lines indicate the mean value at baseline in DAOSD. N = 3–14. One-way analysis of variance was used for statistical testing. Log10 transformation was performed to the raw values after adding a pseudo count (1). NA = not applicable.

To identify differential abundant features (microbial species), we applied the Linear discriminant analysis Effect Size tool^29^ (Fig S3, available at www.ophthalmologyscience.org). In patients with AD, we observed several differentially abundant microbial species across different time points (Fig 8). Most notably, *S. aureus*, a pathogenic microbe associated with AD, was significantly less abundant 1 month (*P* = 0.0250) and 4 months (*P* = 0.0362) after the initiation of dupilumab as compared with baseline (Fig 8A) in the AD group. We observed *S. aureus* in only 2 of 6 patients with DAOSD, without any statistical significance when compared with various visits, and none in control subjects (Fig 8A). In addition, the abundance of *Cutibacterium granulosum* significantly decreased in patients with AD after 4 months as compared with baseline (*P* = 0.0354), whereas it increased in patients with DAOSD during the conjunctivitis episode (Figure 8G). We observed a decreasing trend in terms of abundance of *Liquorilactobacillus mali* in patients with AD and an increasing trend in patients with DAOSD (Fig 8I).

**Figure 9:**
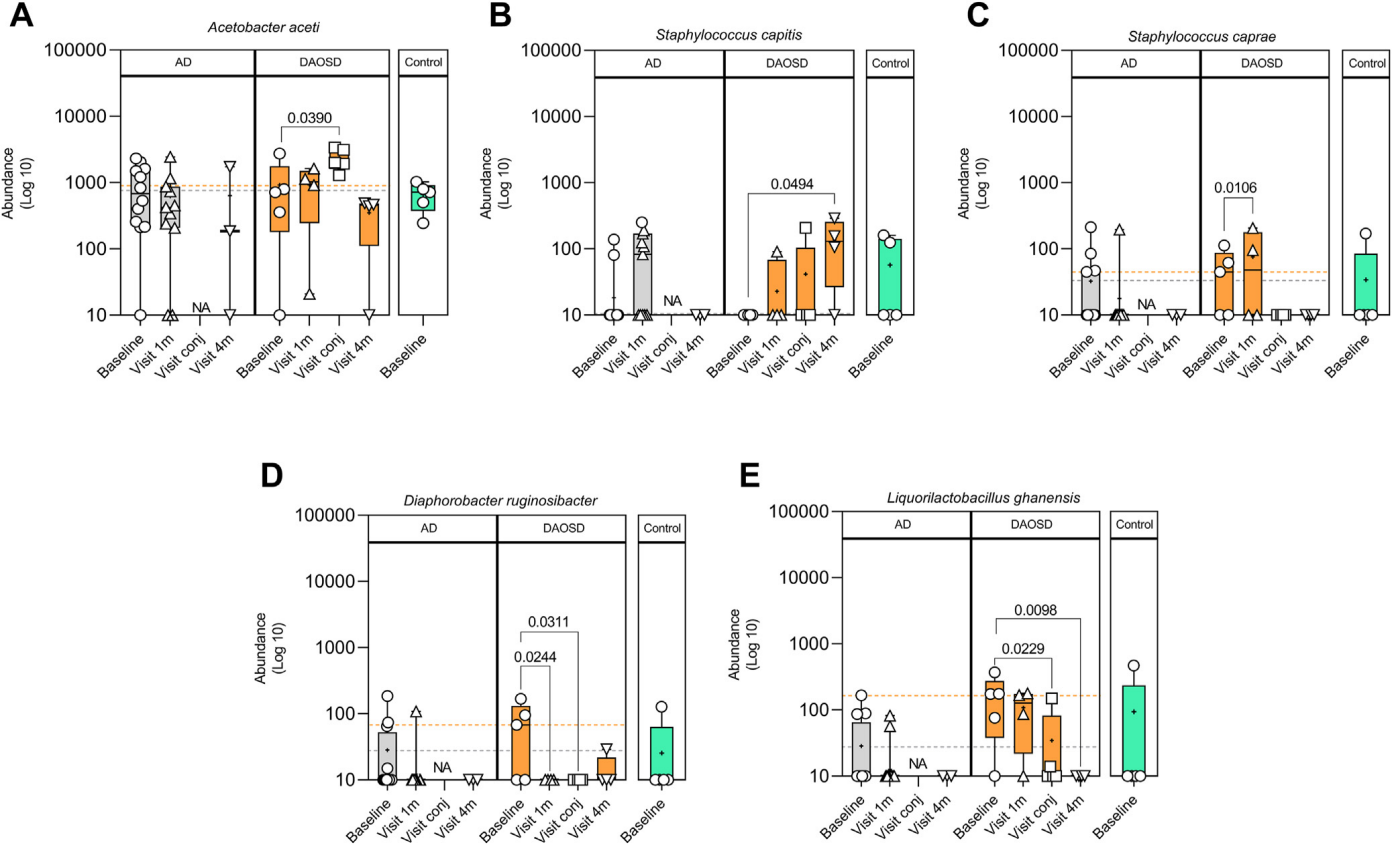
Five bacterial species differ significantly over time on the ocular surface of patients with dupilumab–associated ocular surface disease (DAOSD). **A–E,** Box plot showing various bacterial species in patients with atopic dermatitis (AD), DAOSD, and controls identified by linear discriminant analysis. Data are presented in box and whisker plots showing minimum, maximum, and median values; a diminutive (+) indicates mean values, () gray dotted lines indicate the mean value at baseline in AD, and () orange dotted lines indicate the mean value at baseline in DAOSD. N = 3–14. One-way analysis of variance was used for statistical testing. Log10 transformation was performed to the raw values after adding a pseudo count (1). NA = not applicable.

In patients with DAOSD, performing the linear discriminant analysis enabled the identification of 5 significantly different bacterial species across different time points (Fig 9; Fig S3, available at www.ophthalmologyscience.org). The increased abundance of *A. aceti* was significant during the conjunctivitis episode (*P* = 0.0390 compared with baseline, Fig 9A). In our data set, this was the only species where the abundance was unambiguously higher during the conjunctivitis episode. However, in patients with AD without DAOSD, the abundance of this species remained largely unchanged over time (Fig 9A). In patients with DAOSD, we also observed an increase in the abundance of *Staphylococcus capitis* during the fourth visit as compared with baseline (*P* = 0.0494), whereas this increase was not statistically significant during any visit in patients with AD (Fig 9B). In addition, the abundance of *Staphylococcus caprae* was higher in patients with DAOSD at the 1-month visit as compared with baseline (*P* = 0.0106) and did not change during later visits. In patients with AD, this abundance did not seem to change over time (Fig 9C). The abundance of microbes such as *Diaphorobacter ruginosibacter* (Fig 9D) and *Liquorilactobacillus ghanesis* (Fig 9E), however, decreased over time as compared with baseline.

**Figure 10:**
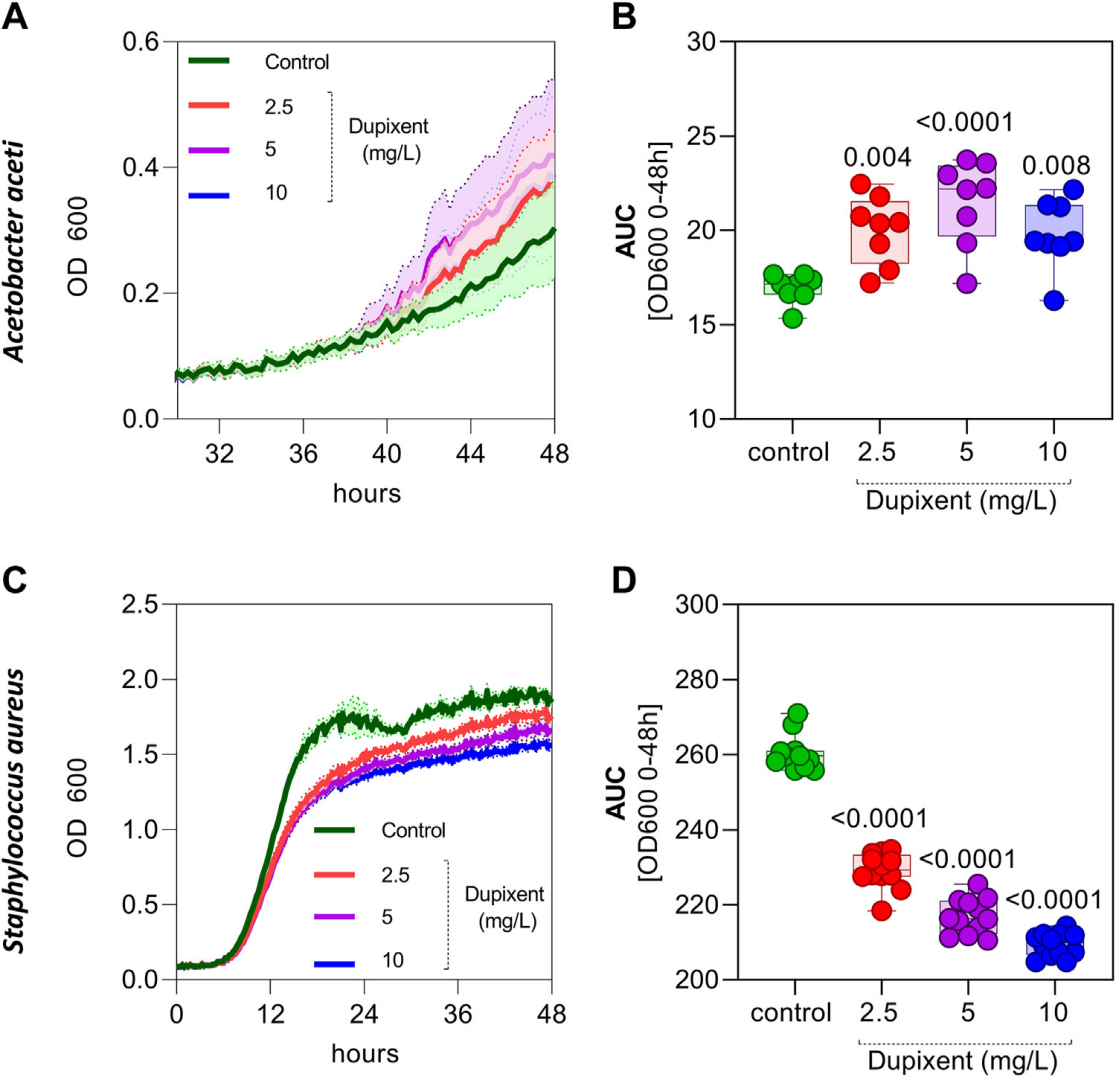
Bacterial growth in presence of dupilumab. *Acetobacter aceti* and *Staphylococcus aureus* were cultured in different concentrations of dupilumab (2.5, 5, and 10 mg/L) and the growth was measured over a 48-hour period. **A,** Growth curves of *A. aceti* in various concentration of dupilumab. **B,** Area under curve (AUC) was calculated from the growth curves from 0 to 48h and data are presented in box and whisker plots showing minimum, maximum, and median values. **C,** Growth curves of *S. aureus* in various concentration of dupilumab. **B,** AUC was calculated from the growth curves from 0 to 48h and data are presented in box and whisker plots showing minimum, maximum, and median values. N = 8–12. One-way analysis of variance was used for statistical testing. *P* values are shown for respective doses vs. control. OD = optical density.

It has been reported that patients with DAOSD have high-dupilumab concentrations in tear fluid.^30^ We hypothesized that certain bacteria, such as *A. aceti*, may survive and adapt to high concentrations of dupilumab and its ingredients, while other bacterial species, such as *S. aureus*, would decrease in abundance. To confirm this hypothesis, we cultured *A. aceti* and *S. aureus* in different concentrations of dupilumab (2.5, 5, and 10 mg/L) and measured growth over a 48-hour period. We observed increased growth of *A. aceti* in a dose-dependent manner, as shown by the growth curves (Fig 10A) and area under the curve (Fig 10B) calculated for 0 to 48 hours. In contrast, *S. aureus* growth decreased in the presence of dupilumab (Fig 10C, D). Overall, we could identify microbial species where the abundance changed over time in AD and DAOSD, and further demonstrate that bacterial species, such as *A. aceti*, could increase in abundance at the ocular surface and contribute to pathogenesis of DAOSD.

### Serum Cytokines

**Figure 11:**
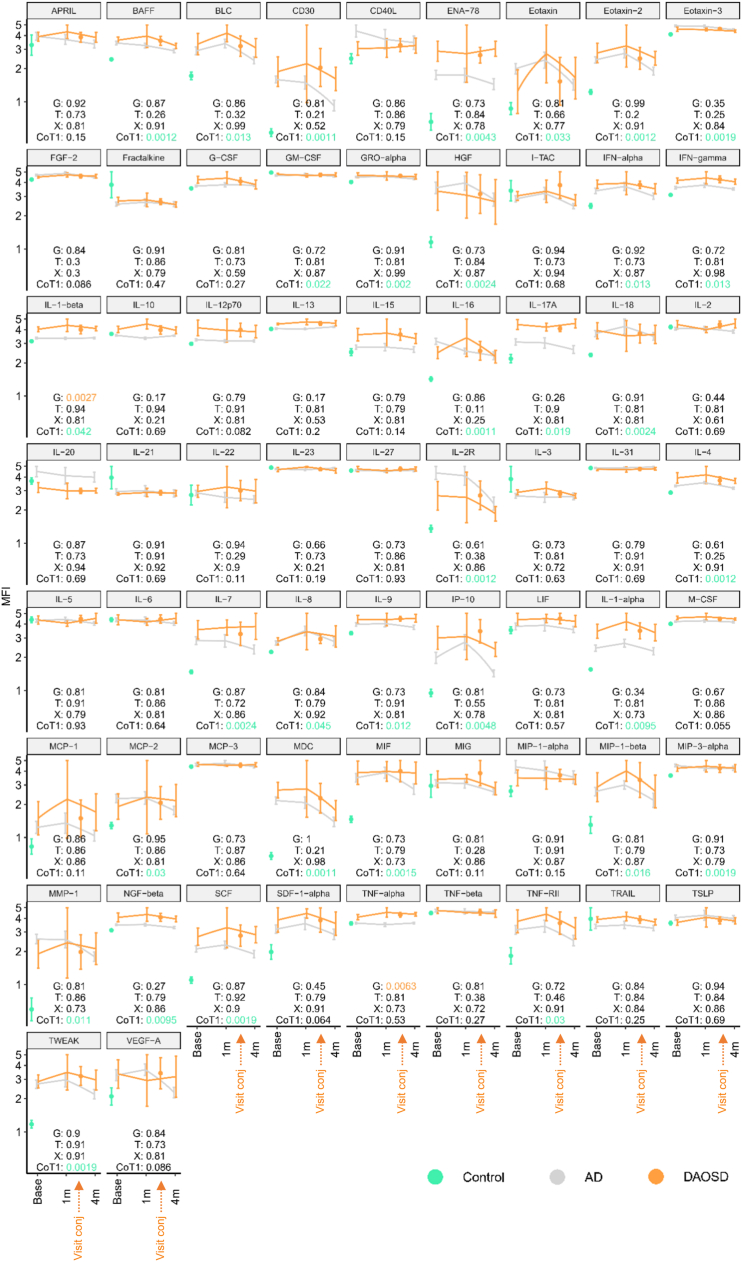
Cytokine profile in serum of patients. The line chart shows the median fluorescent intensity values (group means and 95% confidence limits calculated on the logarithmic scale) of 65 cytokines for controls (at baseline [base]) and for patients with DAOSD and patients without it (at baseline [base], after 1 month [1m] and after 4 months [4m]). All *P* values were false discovery rate corrected for testing 65 cytokines. *P* values < 0.05 were considered statistically significant. The figure shows the details of cytokines with a significant group effect. A Wilcoxon test compared the controls with the patients receiving dupilumab at baseline (CoT1). AD = atopic dermatitis; DAOSD = dupilumab–associated ocular surface disease; G = group effect; IL = interleukin; T = time effect; X = time interaction effect; APRIL = a proliferation-inducing ligand; BAFF = B cell activating factor; BLC = B lymphocyte chemoattractant; CD30 = cluster of differentiation 30; CD40L = cluster of differentiation 40 ligand; ENA-78 = epithelial neutrophil-activating protein 78; FGF-2 = fibroblast growth factor 2; G-CSF = granulocyte colony-stimulating factor; GM-CSF = granulocyte-macrophage colony stimulating factor; GRO-alpha = CXCL1 = C-X-C motif chemokine ligand 1; HGF = hepatocyte growth factor; I-TAC = interferon-inducible T cell alpha chemoattractant; IFN = interferon; LIF = leukemia inhibitory factor; M-CSF = macrophage colony-stimulating factor; MCP = monocyte chemoattractant protein; MDC = macrophage-derived chemokine; MIF = macrophage migration inhibitory factor; MIG = monokine induced by gamma-interferon = CXC ligand 9; MIP = macrophage inflammatory protein; MMP = matrix metalloproteinase; NGF = nerve growth factor; SCF = stem cell factor; SDF = stromal cell-derived factor = CXCL12; TNF = tumor necrosis factor; TRAIL = tumor necrosis factor related apoptosis inducing ligand; TSLP = thymic stromal lymphopoietin; TWEAK = tumor necrosis factor-like weak inducer of apoptosis.

We calculated the median fluorescent intensity values of 65 cytokines (group means and 95% confidence limits calculated on the logarithmic scale) of controls, AD and DAOSD at baseline, after 1 month and after 4 months, and for patients with DAOSD at time of occurrence (Fig 11). By the false discovery rate method it can be expected that, at most, 5% of the significant tests results are false-positive.^31^ Nonparametric longitudinal data analysis using the R package LDpar separated the repeated measurements into a group effect (G), a time effect (T) and a group by time interaction effect (X). Neither the X nor the T were significant for any cytokine. The figure shows the details of cytokines with a significant group effect. We observed a significant increase in several cytokines, such as B-cell activating factor (BAFF), B lymphocyte chemoattractant (BLC), cluster of differentiation (CD)30, epithelial neutrophil-activating peptide (ENA)-78, eotaxin-1, -2 and -3, interferon, IL-1α and IL-1β, and more in patients with AD compared with control subjects at baseline (Fig 11, false discovery rate green values for CoT1). Our results are in line with previous reports).^32^^,^^33^ Cytokines, such as IL-17 and IL-9, seem to increase over time in the DAOSD cohort, whereas others, such as IL-4, IL-13, IL-2R, interferon gamma-induced protein (IP)-10, macrophage-derived chemikine (MDC), monokine induced by gamma interferon (MIG), and tumor necrosis factor (TNF)- receptor type II, seems to decrease during follow up. Statistical testing showed continuous increase in the levels of the proinflammatory cytokines IL-1β by 22.4% (average of various visits) and TNF-α by 17.8% (average of various visits) in patients with DAOSD as compared with patients with AD without DAOSD over the 3 time points (baseline, visit 1, and visit 4; Fig 11). Overall, we report an altered cytokine profile in patients with AD compared with controls, and some cytokines, such as IL-1 β and TNF-α, may play a crucial role in DAOSD.

## Discussion

The pathomechanisms underlying DAOSD are currently not well understood, and we hypothesized that ocular surface microbiome might play a role. To test this hypothesis, we conducted a prospective single-centre study with 20 adult patients with moderate-to-severe AD; 6 (30%) of these patients developed DAOSD after initiation of dupilumab. This result is consistent with the results of phase III clinical trials in which 5% to 28% of adult patients with AD treated with dupilumab were reported to have developed DAOSD.^3^, ^4^, ^5^ In analyses in a clinical setting, the percentage of DAOSD occurrence in adult patients with AD was reported to be even higher (i.e., up to 62%).^6^ In the 6 patients in our study who developed DAOSD over the 4-month period, we observed dense neutrophilic infiltration in their ocular smears. This infiltration was intriguingly linked to elevated levels of proneutrophilic cytokines IL-1β and TNF-α in serum. Neutrophilic infiltration was also associated with persistent colonization in terms of the abundance and diversity of the ocular surface microbiome, and particularly *A. aceti* and *S. capitis*, in patients with DAOSD.

The composition of the healthy ocular surface microbiome has been shown to change with environment and age, and, to date, no international consensus regarding what a healthy core ocular surface microbiome is exists at all.^26^ We currently know that the ocular surface microbiome is paucibacterial, consisting of approximately 0.06 bacteria per human cell. Recent 16S sequencing analyses have shown that the major phyla in healthy adults are *Proteobacteria*, *Actinobacteria*, and *Firmicutes*, and the major genera in healthy adults are *Corynebacteria*, *Staphylococcus*, *Streptococcus*, and *Propionibacterium*.^27^ Regarding changes in the skin microbiome during dupilumab treatment, studies have shown that the frequency of *S. aureus* decreases and that the frequency of *S. epidermidis* and *S*. *hominis* increases, as does the microbial diversity in lesional and nonlesional skin, which positively correlates with treatment response.^34^, ^35^, ^36^ To our knowledge, no study investigating the ocular surface microbiome in DAOSD has been published. Our study represents an altered microbial landscape in patients with AD treated with dupilumab. At baseline, we observed a higher abundance of *S. aureus* on the ocular surface of patients with AD who did not develop conjunctivitis. Our results also show that dupilumab treatment not only reduces the *S. aureus* load in the skin,^34^, ^35^, ^36^ but also on the ocular surface of patients. Intriguingly, this species was not abundant in patients with DAOSD; instead, microbial species, such as *A. aceti*, were clearly more abundant in patients with DAOSD, and especially during the conjunctivitis episode. Although relatively little is known about this species, we know that it is involved in the conversion of alcohol to acetic acid under aerobic conditions and can dissolve insoluble phosphate.^37^ Therefore, we hypothesize that the increased abundance of *A. aceti* after dupilumab treatment leads to the increased production of acetic acid in the eye, which may be involved in the development of conjunctivitis. That said, microbes, such as *A. aceti*, could metabolize dupilumab and produce higher levels of acetic acid; 1 recent study showed substantial levels of dupilumab in tear fluid.^38^ Sodium acetate, one of the ingredients among others (L-arginine hydrochloride, L-histidine, polysorbate 80, and sucrose) in the dupilumab injection, is known to have antimicrobial activity.^2^ Although most microbes are sensitive to acetate, a few, such as *A. aceti*, are known to be resistant and adapt to high-acetate concentrations.^39^ Indeed, our in vitro results demonstrate that growth of *A. aceti* is increased in presence of dupilumab, whereas growth of *S. aureus* was reduced. Whether sodium acetate administered with injection of dupilumab in patients at a distant site may be transmitted in sufficient quantity to the eye remains to be determined. However, if so, it could explain the decreased microbial diversity and abundance in the AD group, versus a persistent colonization in the DAOSD group by species, such as *A. aceti*. As *A. aceti* was reported to be susceptible to gentamicin,^40^ adding antibiotic coverage to currently proposed DAOSD treatment could improve treatment success.

Transient systemic eosinophilia is commonly observed in patients with AD treated with dupilumab. However, Katsuta et al^41^ reported that patients who developed DAOSD had higher blood eosinophil levels 2 months after starting treatment with dupilumab and higher blood basophil levels 2 to 3 months later than patients who do not develop DAOSD while under treatment. In addition, Ferrucci et al^42^ reported that patients who developed DAOSD or facial redness dermatitis during treatment with dupilumab had higher blood eosinophil granulocyte counts 4 months after treatment initiation. In our patient samples, however, we found no significant differences in blood eosinophil counts between patients with DAOSD and without (data not shown) at baseline or 4 months after treatment initiation. In conjunctival biopsies from patients with DAOSD, Bakker et al^12^ found an increased multicellular stromal infiltrate consisting mainly of T cells and granulocytes, accompanied by a deficiency of conjunctival goblet cells. In contrast, we found high number of neutrophils (but few or no eosinophils) in the conjunctivital smears of patients with DAOSD. This difference might be due to the type of material (tissue biopsies vs. conjunctivital smears) used for the investigation. Moreover, this increase in neutrophilic infiltration is likely related to dupilumab-induced microbiome changes on the ocular surface, as previous reports on allergic conjunctivitis indicate a lower density of neutrophils and eosinophils and higher density of mast cells in infiltrates.^43^^,^^44^ However, the in case of corneal infection the number of infiltrating neutrophils is elevated.^45^ In our study, we continued to observe increased neutrophil counts in the DAOSD group (visit 4m, Fig 3C), even after ocular surface treatments with calcineurin inhibitors. We speculate that this is likely due to the persistent microbial colonization in patients with DAOSD, in contrast to patients without it, where the number and diversity of the ocular surface microbiome decreased after dupilumab treatment.

The cytokine levels we observed in serum from our samples were consistent with those of Bakker et al,^12^ who noted a significant increase in TNF-α among other cytokines in conjunctival biopsies from patients with active DAOSD. Indeed, it has been hypothesized that neutrophil recruitment in the eye could be driven by trapped bacteria or bacterial components, such as lipopolysaccharides.^46^^,^^47^

Overall, the EASI and IGA scores and treatment response found in our study were consistent with results from clinical studies and studies in a clinical setting.^48^ Whether the delayed clinical response seen in patients who developed DAOSD (Fig 1) is related to changes in levels of certain cytokines, such as IL-1β and TNF-α, the constitutive ocular surface microbiome and persistent neutrophilic infiltration, warrants further investigation. That said, a dysbiotic ocular surface microbiome could lead to an altered host immune response, resulting in a vicious cycle causing immunologic stimulation and have systemic implications and alter the treatment response.^49^

### Limitations

The limitations of this study are the overall small sample size. Because the study was conducted during the coronavirus disease 2019 pandemic, we were unable to obtain data during some visits. Conjunctival smears collected during the study likely missed several active and relevant cellular populations in the lamina propria that could have been detected by a conjunctival biopsy. However, due to the invasive nature of sample collection by a biopsy and multiple timepoints, we chose minimally invasive sample collection by taking 1 swab per visit (for microbiome analysis) and only 1 smear, which allowed us to perform hematoxylin and eosin staining (but no other stainings) to examine the cellular infiltrate. Some of the ocular swab samples had to be excluded from microbiome sequencing after performing quality control (because of low-microbial DNA yield or PCR and sequencing errors) and bioinformatic analyses (low-quality reads). That said, during the microbiome analysis, we rarefied all the samples to randomly selected number of reads, and performed diversity metrics that can be calculated independent of sample size in various visits. However, we acknowledge that randomized studies in larger cohorts need to be performed to reproduce the results, and to supply more statistical power. Additionally, ocular cytokine profiling and systemic measurements of quantity and quality of proteins and metabolites should be done to better understand the pathophysiology of the disease. The findings of this study should be interpreted with caution because of its limitations. Although we observed changes in both cellular and microbial levels on the ocular surface, the causative relationship between these changes and DAOSD has not been scrutinized in this study.

Although our results need to be confirmed in large cohort studies, based on our findings, antibiotic coverage might be beneficial in management or prevention of DAOSD in addition to currently proposed preservative-free lubricants, anti-inflammatory therapy, such as corticosteroids and calcineurin inhibitors, and mast cell stabilizers/antihistamines. Because *A. aceti* was reported to be susceptible to gentamicin,^41^ combination treatment of dexamethasone/gentamicin, as widely used in ophthalmology, might be beneficial for DAOSD treatment. Our results indicate the potential role played by the ocular surface microbiome and neutrophilic infiltration in DAOSD. A better understanding of the pathophysiology of DAOSD might open new avenues for preventive and therapeutic strategies.
