# Supplementary material for: Persistent Neutrophil Infiltration and Unique Ocular Surface Microbiome Typify Dupilumab–Associated Conjunctivitis in Patients with Atopic Dermatitis
Source: Ophthalmol Sci. 2023 May 29;4(1):100340. doi: 10.1016/j.xops.2023.100340 (PMC10585475; doi:10.1016/j.xops.2023.100340)
Supplement: Supplementary [file mmc1.pdf]

Supplementary Figure 1: Seasonal effects

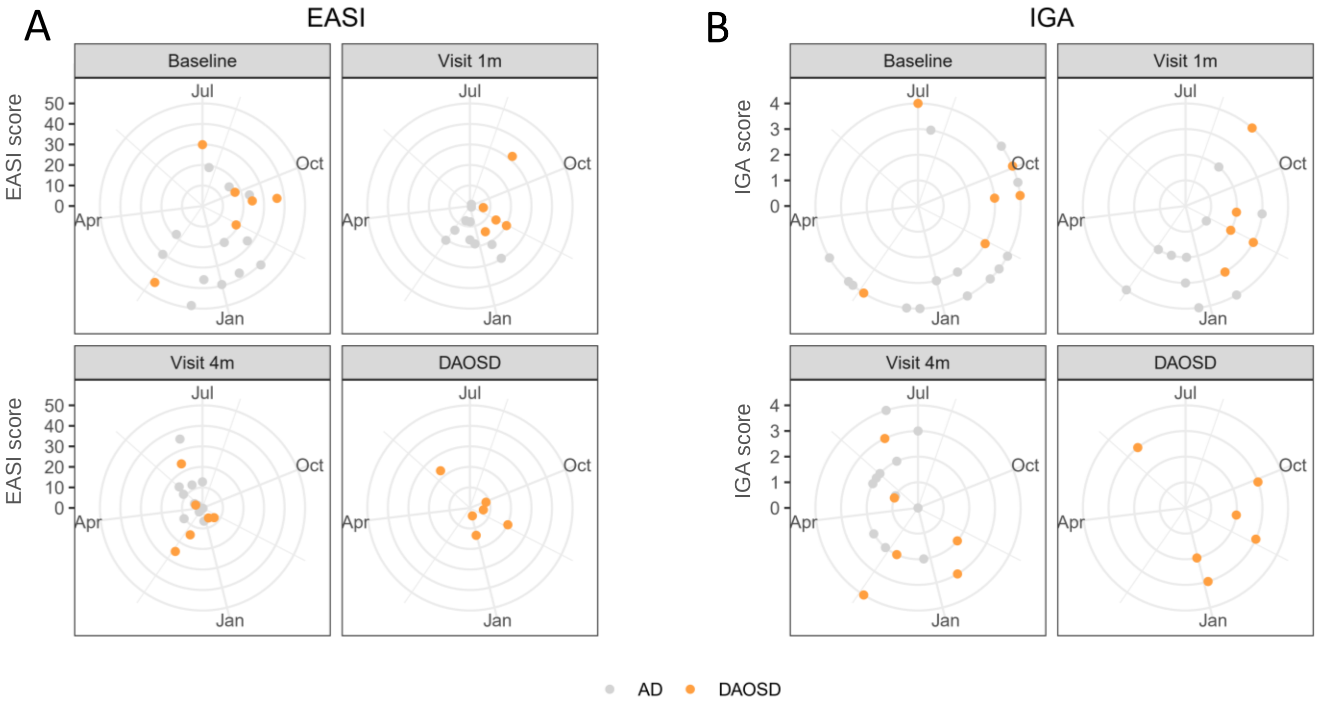

Supplementary Figure 1: Distribution of EASI (A) and IGA (B) dermatological scoring at baseline, 1 month after start of treatment (Visit 1m) and at the end of treatment (Visit 4m) over the year. The scale on left labels the circles of the plot starting at the center.

Supplementary Figure 2: Corelation EASI vs Neutrophils vs Shannon index

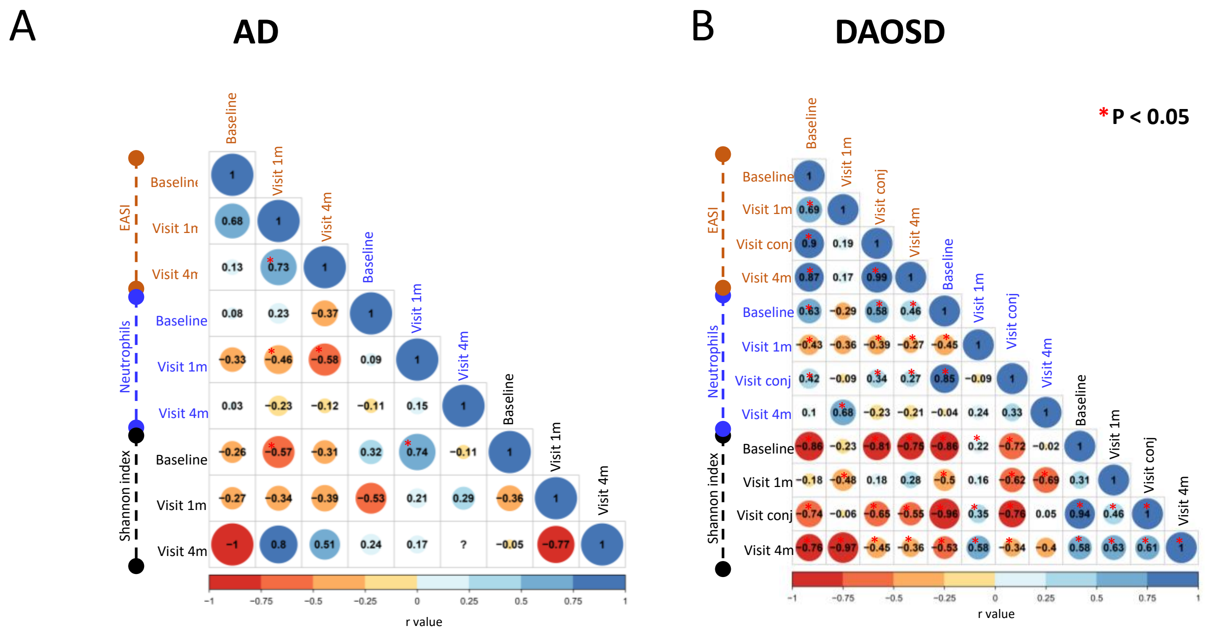

Supplementary Figure 2: Correlation analysis was performed using R studio (RStudio 2021.09.0+351 "Ghost Orchid" Release) and “corrplot” function. Correlation matrix was obtained using variables from EASI scores, ocular surface neutrophil counts and shannon indexes. The values of correlation matrix ranges from -1 to 1 (r value), indicating either a positive or negative correlation. Statistically significant values ($p < 0.05$) are highlighted with *.

Supplementary Figure 3: Linear discriminant analysis (LDA) effect size (LEfSe)

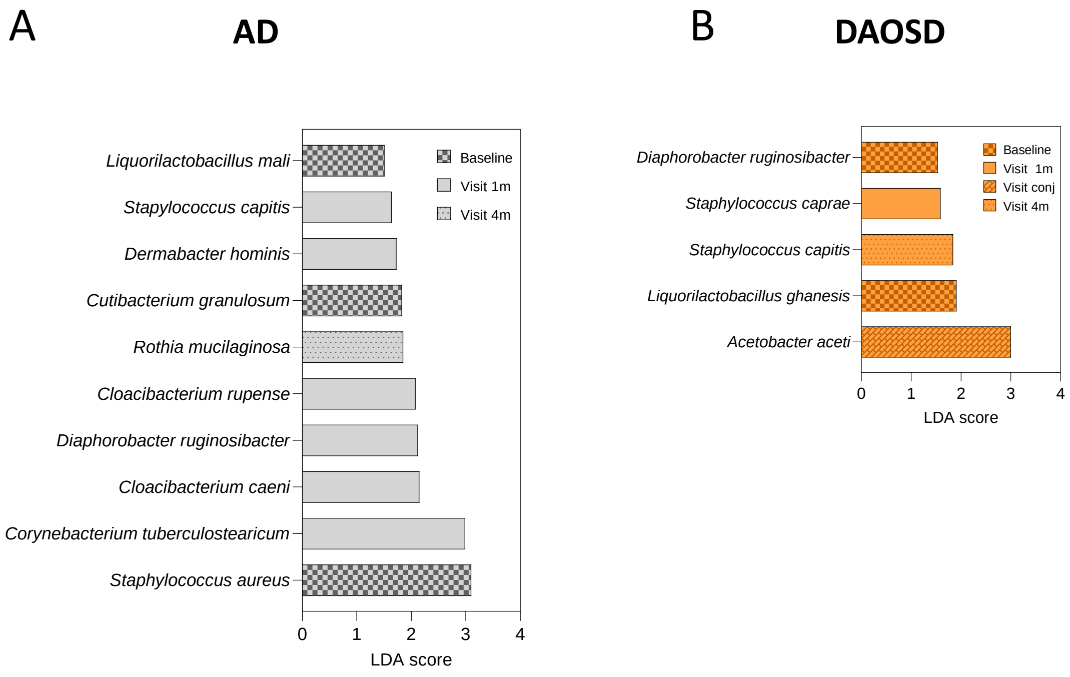

Supplementary Figure 3: LefSE LDA analysis was performed as described in results section of the manuscript. LDA scores are shown here for significantly enriched microbial species in (A) AD and (B) DAOSD at various visits.
